# 

**Published:** 1888-04

**Authors:** 


CONTENTS:

ARTICLE I.—Odonto-Chirurgical Society. By Dr. Williamson ..... ... 529
" II.—Immediate Root Filling. By H. A. Smith, D. D. S ...... 537
" III.—Root Medication. By S. Eschelman, M. D., D. D. S..... 545
" IV.—On Some Cases of Congenital Fissures of the Mouth. By J. Bland Sutton, F. R. C. S .................... 549
" V.—Conservation of the Dental Pulp. By J. R. Callahan, D. D. S .......................... 554
" VI.—Tumors of the Mouth and Jaws. By A. E. Baldwin, M. D., D. D. S............ ...... 558
" VII.—Intermittent Pressure. Its Relation to Orthodontia. By Levitt E. Custer, B. S., D. D. S ................ 563

EDITORIAL, ETC.

The Forty-Eighth Annual Commencement of the Baltimore College of Dental Surgery....... ................. ................. 569
The Sixth Annual Commencement of the Dental Department of the University of Maryland......... ......... ............ 571

MONTHLY SUMMARY.

A Cheap Matrix.................................... ........................ ..... ..... 572

Lead or Amalgam Instead of Gold...... ............. ............ 573

How to Cut a Bottle.................. ............. ...................................... 574

Water at Meals.............................................. .............................. 574

Uses of Chlora-Percha.......................................... ........................ 575

Irregularities... ............. .... ...................................... ................. 575

A Physician and the Toothache ..... .......................................... ......... 576

The Most Convenient Matrix................. ............................... .......... 576

